# The Irish DAFNE Study Protocol: A cluster randomised trial of group versus individual follow-up after structured education for Type 1 diabetes

**DOI:** 10.1186/1745-6215-10-88

**Published:** 2009-09-23

**Authors:** Seán F Dinneen, Mary Clare O' Hara, Molly Byrne, John Newell, Lisa Daly, Donal O' Shea, Diarmuid Smith

**Affiliations:** 1 Department of Medicine, Clinical Science Institute, National University of Ireland, Galway, Ireland; 2 Endocrinology and Diabetes Day Centre, University Hospital Galway, Galway, Ireland; 3 School of Psychology, National University of Ireland, Galway, Galway, Ireland; 4 Clinical Research Facility, National University of Ireland, Galway, Galway, Ireland; 5 St Vincent's University Hospital, Elm Park, Dublin 4, Ireland; 6 Beaumont Hospital, Dublin 9, Ireland

## Abstract

**Background:**

Structured education programmes for individuals with Type 1 diabetes have become a recognised means of delivering the knowledge and skills necessary for optimal self-management of the condition. The Dose Adjustment for Normal Eating (DAFNE) programme has been shown to improve biomedical (HbA_1c _and rates of severe hypoglycaemia) and psychosocial outcomes for up to 12 months following course delivery. The optimal way to support DAFNE graduates and maintain the benefits of the programme has not been established. We aimed to compare 2 different methods of follow-up of DAFNE graduates in a pragmatic clinical trial delivered in busy diabetes clinics on the island of Ireland.

**Methods:**

Six participating centres were cluster randomised to deliver either group follow-up or a return to traditional one-to-one clinic visits. In the intervention arm group follow-up was delivered at 6 and 12 months post DAFNE training according to a curriculum developed for the study. In the control arm patients were seen individually in diabetes clinics as part of routine care. Study outcomes included HbA_1c _levels, self-reported rates of severe hypoglycaemia, body weight and measures of diabetes wellbeing and quality of life. These were measured at 6, 12 and 18 months after recruitment. Generalisability (external validity) was maximised by recruiting study participants from existing DAFNE waiting lists in each centre, by using broad inclusion criteria (including HbA_1c _values less than 13 percent with no lower limit) and by using existing clinic staff to deliver the training and follow-up. Internal validity and treatment fidelity were maximised by quality assuring the training of all DAFNE educators, by external peer review of the group follow-up sessions and by striving for full attendance at follow-up visits. Assays of HbA_1c _were undertaken in a central laboratory.

**Discussion:**

This pragmatic clinical trial evaluating group follow-up after a structured education programme has been designed to have broad generalisability. The results should inform how best to manage the well educated patient with Type 1 diabetes in the real world of clinical practice

**Trial registration:**

Current Controlled Trials ISRCTN79759174

## Background

Type 1 diabetes is a challenging condition to manage. Once diagnosed it requires active patient involvement in self-management of lifestyle issues including diet, physical activity and stress reduction. Most patients are expected to self-administer insulin subcutaneously several times a day and monitor its effects through frequent (and painful) self-monitoring of capillary blood glucose levels. Even in motivated patients it can be difficult to avoid day-to-day fluctuations in blood glucose levels resulting in symptomatic hyper- or hypoglycaemia. Although the risk of chronic microvascular complications of diabetes can be reduced by maintaining good glycaemic control, in an individual patient there is no guarantee that their genetic predisposition will not result in premature impairment of vital organs including the eyes, kidneys and peripheral nerves. Worry about complications and the fear of hypoglycaemia are significant burdens for many people living with the disease [1].

In Ireland and the UK systems for delivering care to individuals with type 1 diabetes have traditionally been very hospital and healthcare professional oriented. Typically a patient will be offered 3-4 visits per year to a hospital outpatient clinic or Diabetes Centre. During these visits a doctor and/or nurse will review certain clinical (e.g., weight, blood pressure) and laboratory (e.g., glycated haemoglobin, lipid levels) parameters and may establish targets for the patient to achieve. The patient's self-monitoring of blood glucose data may be reviewed although this does not always happen [2]. During at least one of these visits annual screening for microvascular complications will be undertaken. Traditionally, education to support self-management has not been a major focus of the diabetes clinic visit. If it is delivered the education is often undertaken in an ad hoc manner and without reference to a clear education plan. Perhaps not surprisingly, when formally assessed through clinical practice audit, outcomes associated with this traditional method of diabetes care delivery are not good. This is true both for adults and children with Type 1 diabetes [3,4] where average levels of HbA_1c _of 8.6 to over 9.5 percent have been reported.

The concept of therapeutic patient education was first introduced to medical practice through the pioneering work of Jean-Phillipe Assal, a Swiss doctor and educationalist [5]. It aims to provide a more holistic approach to patient care while ensuring that the knowledge, skills and attitudes necessary to achieve effective self-management (or mastery) of the long-term condition are delivered to the patient. In the case of Type 1 diabetes a German diabetologist, Michael Berger, and his group in Düsseldorf first operationalised this concept through the development of an "Insulin Treatment and Teaching Programme" in the late 1970's. They demonstrated through randomised controlled trials that this approach was associated with improved glycaemic control and no increase in rates of severe hypoglycaemia [6]. The approach is now widespread throughout Germany [7]. In the late 1990's a group of UK diabetes care professionals observed the Düsseldorf programme and adapted it for delivery in an outpatient setting in 3 UK centres. The Dose Adjustment for Normal Eating (or DAFNE) programme is a 5 day structured education programme covering all aspects of living with diabetes with an emphasis on the key skill of estimating carbohydrate intake and matching insulin to food [8]. Using a waiting list controlled design among individuals with poorly controlled diabetes the UK group showed significant improvement in HbA_1c _levels at 6 months but a diminution of this effect by 12 months. Rates of severe hypoglycaemia did not increase with the improvement in glycaemic control while patient-reported quality of life improved significantly [9].

The DAFNE approach to diabetes care delivery was greeted with considerable enthusiasm by patients [10], healthcare professionals [11] and policy makers [12]. Since publication of the results of the DAFNE trial a further 74 diabetes teams in the UK and Ireland have undertaken training of their staff to deliver the programme [8]. Preliminary reports of audit data from a number of these DAFNE centres suggest that improvements in glycaemic control and psychosocial measures of wellbeing similar to the DAFNE trial are being achieved in routine practice [13]. However, unlike the situation in Germany where sustained benefit has been reported [7], long-term data from the UK indicate a diminution of the HbA_1c _improvement [14]. This raises the issue of how best to support the long-term needs of a patient who has been through DAFNE training. This is the central question being addressed in the Irish DAFNE Study. The hypothesis on which the study is based is that group follow-up of DAFNE graduates is superior to individual follow-up. The philosophy of the DAFNE programme is grounded in Therapeutic Patient Education Theory [15]. The group education approach incorporating theories of adult learning has been used in structured education programmes for people with Type 2 diabetes, such as DESMOND [16] and ROMEO [17]. This paper describes the design, setting, interventions and outcomes of the Irish DAFNE Study, an 18 month cluster randomised trial of self-management support among patients with Type 1 diabetes.

### Irish DAFNE Study Objectives

1. To develop a new model of ongoing care for DAFNE graduates based on structured group follow-up and peer support

2. To undertake an evaluation of this new model of care (group follow-up of DAFNE graduates) comparing it with "usual care", i.e., a return to the standard one-to-one clinic visits following DAFNE training.

## Methods

### Design

**Figure 1:**
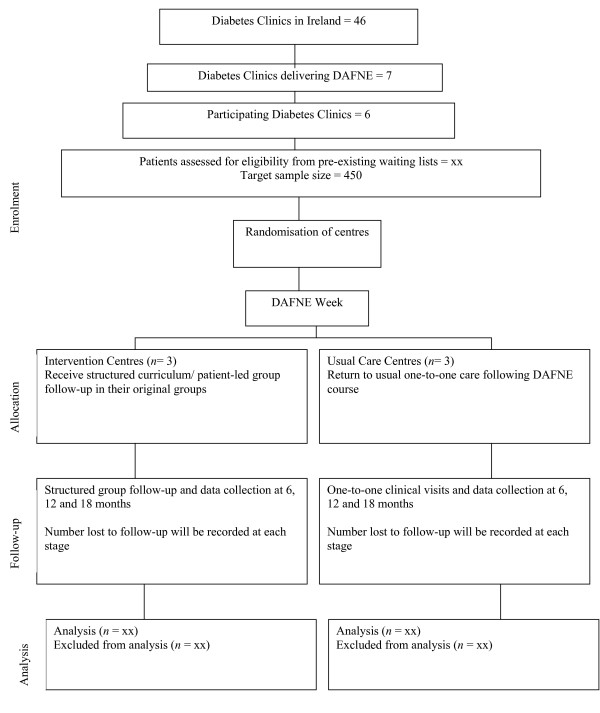
**Flow of centres and participants throughout the trial**.

The Irish DAFNE Study is a pragmatic, open, cluster randomised, parallel group trial comparing 2 different methods of follow-up of patients with Type 1 diabetes who have received the 5-day DAFNE programme in participating Irish hospitals. The study design and patient recruitment are represented in Figure 1, using the CONSORT flow diagram. The CONSORT approach will be followed in reporting the results of this clinical trial [18].

Ethical approval was obtained from the Research Ethics Committee of National University of Ireland, Galway (06/MAY/04), from the Research Ethics Committee of Galway University Hospitals (CA 19), from COREC Northern Ireland (06/NIR01/126) and from the local research ethics committee of each participating hospital. The decision to obtain ethical approval from each participating centre was based on the Irish legislature's interpretation of European Union clinical trials legislation. Had the intervention been a medicinal product (rather than an educational package) this requirement would have been waived in favour of approval from a single Research Ethics Committee.

Written informed consent was obtained from all participants in the Irish DAFNE Study after they had sufficient time to consider the Patient Information Sheet and had any questions relating to their participation answered by study personnel.

### Setting and Centre Recruitment

**Figure 2:**
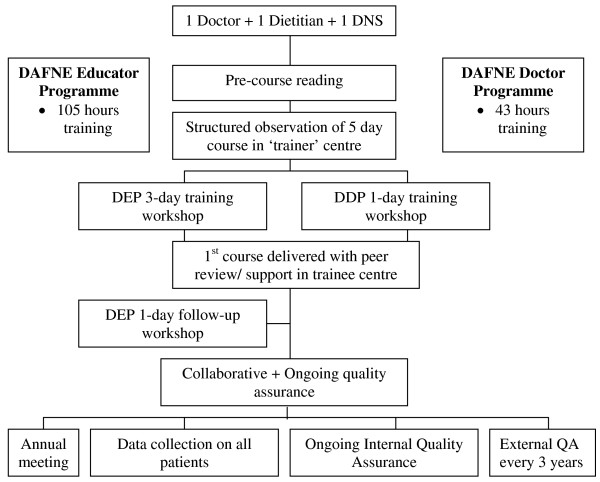
**Becoming a DAFNE Centre**. DNS - Diabetes Nurse Specialist; DEP - DAFNE Educator Programme; DDP - DAFNE Doctor Programme.

There are approximately 35 hospitals in the Republic of Ireland delivering outpatient care to individuals with Type 1 diabetes. The equivalent number for Northern Ireland is approximately 11 hospitals. In 2005 when this study was conceptualised only 2 of these 46 hospitals was offering the DAFNE programme to its patients. An additional 5 diabetes teams were in the process of receiving DAFNE training or had plans to do so in the near future. One of these centres was based in primary care and the lead doctor declined an invitation to participate in the study. The remaining 6 hospital-based teams agreed to combine their efforts into delivering a programme of research, the Irish DAFNE study. Although random selection of diabetes centres would have been preferable this was not an option because of the considerable commitment required on the part of a diabetes team to become a DAFNE Centre (see Figure 2).

### Randomisation and Patient Recruitment

In the study we used a cluster randomisation with the unit of randomisation being the participating DAFNE centre. There are 6 participating centres in this study and it was anticipated that each cluster would have approximately 75 participants each. We felt that DAFNE educators would not be able to separate the 2 approaches to follow-up if they were expected to deliver both. By randomising centres to deliver one or other method of follow-up we hoped to minimise or avoid contamination between the two arms of the study. The process of randomisation of centres was undertaken by a statistician not involved in the study and who was blind to the identity of the hospitals being randomised. The randomisation was done using a computer-generated table of random numbers.

Prior to the study each of the participating DAFNE centres used a similar approach to filling DAFNE courses. A waiting list was maintained of individuals with Type 1 diabetes attending the centre who had expressed an interest in receiving DAFNE training. Using this list, groups of approximately 50 patients were invited to attend a recruitment evening in which the DAFNE course was described and members of the DAFNE team were available to answer questions. A brief description of the study aims and objectives was included in the material presented at the recruitment evening. Individuals were asked to consider participating in the study but were not required to give an answer at the recruitment evening. Following the recruitment evening approximately 6 DAFNE courses (each comprising 8 participants) would be filled. Prior to the week of DAFNE training, individuals were invited to attend a pre-course assessment visit. During this visit a DAFNE educator would make sure that the individual met the inclusion criteria for the study. Informed consent was generally obtained at this visit and baseline study questionnaires were given to the patient.

Study Inclusion criteria:

1. At least 18 years of age at recruitment

2. Diagnosed with type 1 diabetes for at least one year

3. Attending the adult diabetes clinic in one of the participating centres

4. The ability to speak and read English

5. A willingness to monitor blood sugar levels at regular intervals

6. A willingness to transition to a basal/bolus insulin regimen prior to DAFNE training (if not already on such a regimen)

7. A glycosylated haemoglobin (HbA_1c_) level below 13 percent at recruitment.

Study Exclusion criteria:

1. Diagnosed with type 2 diabetes

2. Attending a paediatric clinic

3. Pregnant or planning a pregnancy in the next 2 years

4. The presence of advanced diabetes complications (e.g. kidney failure with serum creatinine >250 μmol/L)

5. Serious co-morbidity likely to interfere with study participation (assessed by the study centre's physician)

6. Previous DAFNE training or current use of a continuous subcutaneous insulin infusion pump

### DAFNE and Study Interventions

The DAFNE course is delivered over 5 consecutive days and involves 38 hours of structured education in all aspects of managing Type 1 diabetes. The education is delivered by a Diabetes Specialist Nurse, Dietitian and Doctor all of whom have undergone training in delivery of the DAFNE curriculum. Figure 2 illustrates the rigorous training process that a diabetes team has to go through to become a DAFNE centre. One of the real strengths of the DAFNE programme is the quality assurance that is incorporated into its delivery. DAFNE educators regularly undergo peer review (both within their centre and via external peer review) to ensure they are maintaining a consistent high standard of course delivery. This means that the basic package (the DAFNE course) delivered in each of the participating DAFNE centres was uniform and standardised to a level appropriate for a research study. Details of the individual sessions that comprise the DAFNE course are available on the DAFNE Collaborative website [8] and all resource literature is available to DAFNE graduates via the DAFNE Online website [19]. Participants in the intervention arm centres also receive sessions on goal setting and action planning as part of the DAFNE week.

Following DAFNE course delivery patients are invited back as a group for a 6 week return visit with the educators (nurse and dietitian) who delivered their original course. This was considered an essential component of the basic DAFNE course and was not removed from either arm of the study. After this 6 week return visit participants in the usual care arm of the study are offered return visits to the diabetes clinic at 6, 12 and 18 months post DAFNE training. This reflects usual care in Irish DAFNE centres prior to the study. The protocol does not stipulate what issues are covered in these visits. The only stipulation is that participants should not be seen in a group setting. It is recognised that attempts are likely to be made to have patients in this arm of the study seen by the doctor, nurse and/or dietitian involved in their original DAFNE training. However, patients may also be seen by diabetes team members not trained in DAFNE.

Participants in the intervention arm of the study are also seen at 6, 12 and 18 months. The 6 and 12 month visits are organised as group education sessions and build on the concepts of goal setting and action planning introduced during the original DAFNE course. A follow-up curriculum was developed to guide DAFNE educators involved in delivering these sessions and specific training in the delivery of this curriculum was given to all intervention arm DAFNE educators. Groups are encouraged to determine their own priorities and select from a range of DAFNE self-management skills review topics designed specifically for these follow-up sessions. The role of the DAFNE educator is to achieve a blend of a patient-centred and curriculum-centred approach to the session. Follow-up curriculum topics include HbA_1c _and targets, diary keeping and self-monitoring, principles of dose adjustment, carbohydrate calculation, hypoglycaemic management, exercise and physical activity, alcohol and sick day rules. An example of a typical timetable for a group follow-up session is outlined in Table 1. Where DAFNE graduates are unwilling or unable to participate in a follow-up session with their original group they are offered a follow-up session with a different DAFNE group from that centre. Every effort is made to avoid delivering follow-up on a one-to-one basis in this arm of the study. In keeping with an emphasis on quality assurance an external peer review of the delivery of one 6 month follow-up session in each of the 3 intervention arm centres will be undertaken during the study.

**Table 1:** Typical timetable used during the intervention follow-up sessions

09:30	Welcome
09:40	HbA_1c _results, review of blood sugar and targets

10:10	Quiz to identify 3 areas the group would like to focus on (patient-centred approach)

10:20	Curriculum used to guide group-led discussion of identified topics

12:10	Goal setting and action planning

12:30	Close

### Outcomes and Measurement

The primary outcome (on which sample size calculations are based) is the change in HbA_1c _between baseline and the 18 month follow-up visit. Patient-reported rates of severe hypoglycaemia (defined as a hypoglycaemic episode requiring the assistance of another person for treatment) will be reported as a secondary outcome along with change in weight and in psychosocial measures of wellbeing and quality of life.

Table 2 shows the measurement plan for the study. Because of the potential for inter-laboratory variation in the method and reporting of HbA_1c _results this outcome variable was assayed centrally. Blood was sent to the Royal Victoria Hospital in Belfast which has a track record of co-ordinating laboratory measurements for large scale studies. The method used was a DCCT-aligned HPLC assay (ADAMS-A_1c _HA-8160). Lipid levels were measured in the local laboratory in each hospital. License agreements were obtained for all questionnaires used in the study. The **Diabetes-Specific Quality of Life Scale (DSQOLS) **was originally developed in Germany [20] and is a quality of life measure specific for people with type 1 diabetes. It consists of 10 goal or preference items that are weighted to 10 satisfaction items resulting in a preference-weighted treatment satisfaction score. A further 57 items form 8 domains and are rated on a 6-point Likert scale with a total score reflecting quality of life. The domains are social relations, leisure time flexibility, physical complaints, worries about the future, diet restrictions, daily hassles, fear of hypoglycaemia and daily burdens & restrictions. For use in the Irish DAFNE Study the scale was altered slightly from the original to reflect linguistic differences and more culturally appropriate scale-items. These slight modifications did not alter the psychometric properties of the instrument [21]. The **Problem Areas in Diabetes (PAID) **measure is a 20 item questionnaire that uses a Likert-scale to assess an individual's diabetes-related distress [22]. The **Hospital Anxiety and Depression Scale (HADS) **is a dual instrument that uses 7 items along a Likert-scale to measure both anxiety and depression [23]. It is a suitable self-rating scale for anxiety and depression in patients with both somatic and diagnosed mental health issues with good reliability and responsiveness [24].

**Table 2:** Data collections and follow-up intervals

	**Baseline**	**6 month**	**12 month**	**18 month**
**Weight**	x	x	x	x

**Central HbA**_**1c**_	x	x	x	x

**Lipid panel**	x	x	x	x

**QOL measures (DSQOLS, EQ-5D, PAID, HADS)**	x	x	x	x

**Rate of severe hypoglycaemia**	x	x	x	x

DSQOLS - Diabetes-Specific Quality of Life Scale; PAID - Problem Areas in Diabetes; HADS - Hospital Anxiety and Depression Scale

The Irish DAFNE Study also includes a qualitative sub-study and a health economic analysis. The qualitative research involves in-depth interviews of a sub-set of participants at 6 weeks, 6 months and 12 months after delivery of the DAFNE course. The aim of this work is to explore participants' attitudes towards DAFNE and the different methods of follow-up being evaluated in the main study. The health economic analysis will identify, measure, value and compare the costs and outcomes of the 2 different methods of follow-up of DAFNE graduates and examine both of these relative to usual care. A health economic questionnaire has been developed specifically for the study and this is administered at baseline and at 6, 12 and 18 months following DAFNE training.

### Statistical Considerations

#### Sample Size

We used a sample size calculator designed for cluster randomised trials [25]. We based the sample size calculation on an anticipated HbA_1c _difference between the 2 arms of the study of 0.5 percent from month 6 onwards. This came from the observation in the UK study that DAFNE training led to a 1.0 percent drop in HbA_1c _at 6 months but reverted to 0.5 percent by 12 months [9]. Our study hypothesis is that group follow-up will be able to maintain the benefit out to 18 months. Unlike the UK DAFNE Study patients were not excluded with baseline HbA_1c _below 7.5 percent. Based on a standard deviation of HbA_1c _of 1.2 and an intra-class correlation co-efficient of 0.05 we estimated that 450 patients would be required to detect a 0.5 percent difference with 90 percent power.

#### Planned Analyses

Initially, descriptive analysis will be conducted to fully profile both the study centres (number of educators and doctors, years since initial training and size of DAFNE waiting list) and the participants (age, gender, years since diagnosis, baseline HbA_1c_, weight, lipid levels and socio-economic status). The main analysis, for the entire cohort, will be an intention-to-treat analysis and will compare differences between HbA_1c _at baseline and at 18 months in the 2 treatment arms, adjusting for age, gender, study centre and years since diagnosis. Changes in the rate of severe hypoglycaemia will also be examined.

In addition to this intention-to-treat analysis we will also undertake a "per protocol" analysis on those individuals who attended all of the educational sessions in the intervention arm.

Sensitivity analyses will explore whether adherence to the intervention influences the effect of the intervention on primary outcome.

#### Sub-group analyses

For those individuals with HbA_1c _above and below 7.5 percent at baseline a separate intention-to-treat analysis will be conducted to compare differences between HbA_1c _at baseline and at 18 months in the 2 treatment arms. Changes in the rate of severe hypoglycaemia will also be examined for those with baseline HbA_1c _above and below 7.5 percent at baseline. The rationale for this approach is that "benefit" from DAFNE training for individuals with low HbA_1c _may amount to a reduction in the frequency of severe hypoglycaemia.

#### Psychosocial analyses

With regard to the psychosocial measures, suitable numerical and graphical summaries (e.g. box plots, scatterplots and case profile plots) will be presented to summarise the within-subject (i.e. time) and between-subject (i.e. treatment group) factors.

Several analyses will be performed to compare the change in the psychosocial response variables across time and between groups. These will include linear mixed models initially, using baseline as a covariate and subsequently where the response variables will be expressed as changes from baseline. Different covariance structures will be compared in order to best model the correlation structure within subject.

An intention to treat analysis will be performed to compare the estimated effect of the psychosocial response variables when imputing values for all missing data to the results obtained when analysing the data while ignoring missing data.

Multiple imputations [26,27] will be performed using a Predictive Model Based Method where each missing value is replaced by 5 imputed values. The imputations will be generated via randomly drawn regression model parameters from the Bayesian posterior distribution based on the cases for which the imputation variable is observed.

An estimate of the difference in the mean response, for each of the psychosocial response variables of interest, when comparing those on the intervention to the controls, will be provided by pooling the results of each 'complete' model (i.e. with imputed values) using the Barnard-Rubin adjustment method [28]. The estimated coefficients, standard errors and p-values for the intervention to control comparison from the original and complete analyses will be reported for each of the psychosocial response variables in addition to the fraction of information about the coefficients missing due to non-response/non-attendance.

#### Trial Governance

The co-ordinating centre for the study is in the Diabetes Centre in University Hospital Galway. The Principal Investigator and the Project Manager are based here. Each participating centre has a local Principal Investigator and a lead DAFNE Educator. As well as reporting to the Project Manager on matters relating to the running of the study each DAFNE centre also has a reporting arrangement with the Central DAFNE Co-ordinating centre in Northumbria NHS Trust in North Tyneside, UK. Central DAFNE maintains the DAFNE database, an internet-based, password-protected database used to store demographic, clinical and laboratory data on DAFNE graduates throughout the UK and Ireland. Irish centres enter data onto the DAFNE database. The Project Manager is able to access data from Irish centres and undertakes quality assurance of these data. Requests are made to Central DAFNE for anonymised data downloads to enable quality assurance, data cleaning and statistical analyses to be undertaken.

A Steering Group advises on any issues that arise relating to the smooth running of the study. In March 2009 the funding agency (the Health Research Board) undertook a rigorous mid-term review of the study. This mid-term review panel included international experts in diabetes education research, health services research and clinical trial methodology. The Steering Group responded to a number of issues raised by the panel and received sign-off and a guarantee of ongoing funding to study completion in August 2010.

## Discussion

Diabetes education to support self-management is widely accepted as an integral component of good diabetes care. Despite this, it is only recently that attention has been paid in the UK to what constitutes effective diabetes education [12,29]. The term structured education has been introduced to define programmes of diabetes education that have certain key elements including a curriculum, trained educators, quality assurance of the delivery of the programme and audit of outcomes of the education. Systematic reviews of diabetes education studies have demonstrated a need for better definition of the intervention and for longer term follow-up or "booster" education after the initial session [30,31]. The American Diabetes Association publishes and regularly updates a set of Standards for Diabetes Self-Management Education [32]. In its January 2009 update the Taskforce on Diabetes Self-Management Education (DSME) included as one of its guiding principles the statement that "ongoing support is critical to sustain progress made by participants during the DSME program". A recent framework document from Australia has outlined in a very comprehensive manner the outcomes that would be expected to be impacted by diabetes education [33,34]. The authors point out that those outcomes that are most likely to be impacted upon by education (knowledge, understanding, self-determination, psychological adjustment) are difficult to measure while those clinical outcomes that are easier to measure are less likely to be directly impacted upon by education.

Given all of these difficulties and a lack of a clear consensus on diabetes education in Ireland, a group of Irish diabetes centres has received training and begun delivering a high quality structured education programme, DAFNE. The Irish DAFNE study, described in detail in this report, will evaluate 2 different approaches to supporting DAFNE graduates in implementing and maintaining good self-management skills in the 18 months after their initial diabetes education. The results of this study should inform future policy on diabetes education in Ireland. The results will also inform the important scientific question of how best to provide diabetes self-management support. The qualitative and health economic research being undertaken within the Irish DAFNE Study will give a more in-depth perspective on the impact of this approach at the level of the individual patient and at the level of society.

The design of the Irish DAFNE Study presented several challenges to the study steering group. Although guided by the CONSORT statement on the conduct and reporting of high quality randomised controlled trials [18], it became clear that this was not a straightforward randomised controlled trial. The Irish DAFNE Study is evaluating a non-pharmacological treatment intervention using a cluster randomised design and has several features of a pragmatic trial. Therefore, 3 of the extensions of the CONSORT statement are relevant to our study and its description [35].

A number of design features help with the study's internal validity (i.e., they minimise bias). These include the uniform approach to DAFNE training across centres and the incorporation of peer review and quality assurance into the delivery of the programme. The central measurement of HbA_1c_, one of the main study outcomes, is also a strength of the design. In terms of external validity (or generalisability) the study also has strengths including the recruitment of participants from existing waiting lists in each centre, the use of broad inclusion criteria for potential participants and the delivery of the intervention by diabetes clinic personnel. A potential weakness (in terms of generalisability) is the fact that the results could be seen as relevant only to centres delivering structured group education. However, including DAFNE and other similar programmes there are now in the region of 150 Diabetes Centres across the UK and Ireland involved in this type of activity.

Most importantly perhaps, the results of the Irish DAFNE Study will be relevant to individuals living with Type 1 diabetes. At a time when more and more diabetes studies are funded by pharmaceutical companies with the intention of getting a new product to market or identifying a new indication for an existing drug, it is important not to forget about interventions and outcomes that are important to patients. A recent review of ongoing diabetes studies (identified through clinical trial registries) found that only 18 percent included patient important outcomes as their primary outcome [36]. When primary or secondary outcomes were examined the number including patient-important outcomes increased to 46 percent.

It is not easy to secure funding for large studies of non-pharmacological interventions such as a diabetes education programme. Although these studies are beginning to appear in the Type 2 diabetes literature [16,17] the Irish DAFNE Study will be one of the largest in the area of Type 1 diabetes and should inform the design and reporting of future studies.

## Competing interests

The authors declare that they have no competing interests.

## Authors' contributions

SFD is the principal investigator for the Irish DAFNE Study. DOS and DS are both local lead investigators at the participating centres across Ireland and are members of the Steering Group. JN is the trial statistician. MCOH (previously LD) is the trial project manager. SFD, MCOH, JN and MB drafted the manuscript. All authors read and approved the final manuscript. SFD is the paper guarantor.
